# Alterations in faecal microbiome and resistome in Chinese international travellers: a metagenomic analysis

**DOI:** 10.1093/jtm/taad027

**Published:** 2023-03-02

**Authors:** Man Kit Cheung, Rita W Y Ng, Christopher K C Lai, Chendi Zhu, Eva T K Au, Jennifer W K Yau, Carmen Li, Ho Cheong Wong, Bonnie C K Wong, Kin On Kwok, Zigui Chen, Paul K S Chan, Grace C Y Lui, Margaret Ip

**Affiliations:** Department of Microbiology, Faculty of Medicine, The Chinese University of Hong Kong, Hong Kong Special Administrative Region, China; Department of Microbiology, Faculty of Medicine, The Chinese University of Hong Kong, Hong Kong Special Administrative Region, China; Department of Microbiology, Faculty of Medicine, The Chinese University of Hong Kong, Hong Kong Special Administrative Region, China; Department of Microbiology, Faculty of Medicine, The Chinese University of Hong Kong, Hong Kong Special Administrative Region, China; University Health Service, The Hong Kong Polytechnic University, Hong Kong Special Administrative Region, China; Department of Microbiology, Faculty of Medicine, The Chinese University of Hong Kong, Hong Kong Special Administrative Region, China; Department of Microbiology, Faculty of Medicine, The Chinese University of Hong Kong, Hong Kong Special Administrative Region, China; University Health Service, The Hong Kong Polytechnic University, Hong Kong Special Administrative Region, China; Department of Medicine & Therapeutics, Faculty of Medicine, The Chinese University of Hong Kong, Hong Kong Special Administrative Region, China; The Jockey Club School of Public Health and Primary Care, Faculty of Medicine, The Chinese University of Hong Kong, Hong Kong Special Administrative Region, China; Stanley Ho Centre for Emerging Infectious Diseases, The Chinese University of Hong Kong, Hong Kong Special Administrative Region, China; Hong Kong Institute of Asia-Pacific Studies, The Chinese University of Hong Kong, Hong Kong Special Administrative Region, China; Department of Microbiology, Faculty of Medicine, The Chinese University of Hong Kong, Hong Kong Special Administrative Region, China; Department of Microbiology, Faculty of Medicine, The Chinese University of Hong Kong, Hong Kong Special Administrative Region, China; Department of Medicine & Therapeutics, Faculty of Medicine, The Chinese University of Hong Kong, Hong Kong Special Administrative Region, China; Department of Microbiology, Faculty of Medicine, The Chinese University of Hong Kong, Hong Kong Special Administrative Region, China

**Keywords:** antibiotic resistance genes, doxycycline, antimalarial prophylaxis, alcohol-based hand sanitizer, gut microbiome, antimicrobial resistance, Hong Kong

## Abstract

**Background:**

International travel increases the risk of acquisition of antibiotic-resistant bacteria and antibiotic resistance genes (ARGs). Previous studies have characterized the changes in the gut microbiome and resistome of Western travellers; however, information on non-Western populations and the effects of travel-related risk factors on the gut microbiome and resistome remains limited.

**Methods:**

We conducted a prospective observational study on a cohort of 90 healthy Chinese adult residents of Hong Kong. We characterized the microbiome and resistome in stools collected from the subjects before and after travelling to diverse international locations using shotgun metagenomic sequencing and examined their associations with travel-related variables.

**Results:**

Our results showed that travel neither significantly changed the taxonomic composition of the faecal microbiota nor altered the alpha (Shannon) or beta diversity of the faecal microbiome or resistome. However, travel significantly increased the number of ARGs. Ten ARGs, including *aadA*, TEM, *mgrB*, *mphA*, *qnrS9* and *tetR*, were significantly enriched in relative abundance after travel, eight of which were detected in metagenomic bins belonging to *Escherichia/Shigella flexneri* in the post-trip samples. In sum, 30 ARGs significantly increased in prevalence after travel, with the largest changes observed in *tetD* and a few *qnrS* variants (*qnrS9*, *qnrS* and *qnrS8*). We found that travel to low- or middle-income countries, or Africa or Southeast Asia, increased the number of ARG subtypes, whereas travel to low- or middle-income countries and the use of alcohol-based hand sanitizer (ABHS) or doxycycline as antimalarial prophylaxis during travel resulted in increased changes in the beta diversity of the faecal resistome.

**Conclusions:**

Our study highlights travel to low- or middle-income countries, Africa or Southeast Asia, a long travel duration, or the use of ABHS or doxycycline as antimalarial prophylaxis as important risk factors for the acquisition/enrichment of ARGs during international travel.

## Introduction

The gut microbiome is an important reservoir of antibiotic resistance genes (ARGs).^1^ Understanding the factors that shape the human gut resistome is crucial to facilitate the fight against antimicrobial resistance. International travel is well described to be associated with the acquisition of antimicrobial resistant (AMR) bacteria in the gastrointestinal tract. For instance, up to 20–50% of international travellers were reported to acquire multidrug-resistant (MDR) Enterobacteriaceae while travelling abroad, including extended spectrum β-lactamase-producing Enterobacteriaceae (ESBL-E) and carbapenemase-producing Enterobacteriaceae (CPE).^2^ While most previous studies have focused on these specific opportunistic pathogens or specific ARGs,^3^^,^^4^ a comprehensive risk assessment of AMR needs to include also commensals of the human gut because of their potential to acquire ARGs through horizontal gene transfer.^5^ Besides, recent research has demonstrated the importance of studying the entire gut resistome instead of focusing on just a small number of specific ARGs.^6^

To date, several studies have investigated the effects of international travel on the gut microbiome and/or resistome using either 16S rRNA or shotgun metagenomic sequencing.^6–12^ Some of these studies have revealed travel-related alterations in the gut microbiota composition and/or ARG profiles, whereas some others have reported no significant changes.^6–10^ The majority of these studies was based on Western populations, and the only study on Asian travellers was limited by a small sample size (*n* = 32) and a narrow range of travel destinations (mostly Asian countries).^12^ As a result, our current understanding of the effects of international travel on the gut microbiome and resistome of non-Western populations remains limited. Studies on different ethnic groups are relevant because ethnicity is known to be an important factor affecting both the gut microbiome and ARG profiles.^13^^,^^14^ Besides, whereas travel destination has been identified to affect the acquisition, abundance and diversity of ARGs in international travellers,^6^ the effects of other travel-related risk factors on the gut microbiome and resistome remain to be explored.

Hong Kong is an international travel hub. In 2019 alone, there were over 94 million departures from Hong Kong (http://data.worldbank.org/). The neighbouring countries and regions of Hong Kong have reported higher rates of AMR bacteria.^15^ Here, we conducted a prospective observational study on a cohort of 90 healthy Chinese adult residents of Hong Kong who planned to travel abroad. We characterized their faecal microbiome and resistome before and after travelling to diverse international locations using shotgun metagenomic sequencing and examined their associations with travel-related variables. Our specific objectives were to identify the travel-related risk of acquisition of carriage of AMR bacteria in the gut and determinants of the gut resistome in Chinese travellers.

## Methods

### Study cohort

Healthy subjects who planned to travel abroad were prospectively recruited from students and staff members of The Chinese University of Hong Kong and The Hong Kong Polytechnic University from November 2018 to January 2020. Each participant provided a stool sample and completed a questionnaire before and after travel. Information collected includes subject demographics such as sex and age and travel-related information such as destination and duration (Table S1). Travel destinations were categorized based on the country income according to the World Bank country classifications by income level (http://blogs.worldbank.org/) and geographical regions. Anthropometric parameters such as body weight and height were measured by research staff at the time of recruitment. The inclusion criteria for the current study were ethnically Chinese aged ≥18 years at the time of recruitment. Subjects with gastrointestinal diseases, including malignancy, inflammatory bowel disease and resection of small or large bowel, or who used antibiotics within last three months before travel, except for prophylaxis required for travel, were excluded. All samples were collected before the COVID-19 pandemic broke out. No statistical methods were used to determine the sample size. The study was approved by the Joint Chinese University of Hong Kong—New Territories East Cluster Clinical Research Ethics Committee (CREC 2018.272). Written informed consent was obtained from all participants prior to sample collection according to the Declaration of Helsinki.

### Sample collection, DNA extraction and shotgun metagenomic sequencing

Stool samples were collected from the subjects before and within 1–2 days after return from travel using stool collection kits provided. The samples were stored in their home freezers before pickup and stored at −80°C in the laboratory until further processing. Prior to storage, subsamples were screened for ESBL-E and CPE by culture methods using selective media (CARBA SMART, bioMérieux) as for the surveillance protocol for screening of MDR Enterobacteriaceae. Total DNA was extracted using the DNeasy PowerSoil Pro Kit (Qiagen, Germany) according to the manufacturer’s protocol. DNA library was prepared using the NEBNext Ultra II DNA Library Prep Kit (New England Biolabs, UK) and sequenced on an Illumina NovaSeq platform following the 2 × 150 bp paired-end sequencing protocol.

### Profiling of gut microbiome and resistome

Raw sequence reads were first quality-filtered using Trimmomatic v0.39 with the default parameters, except for MINLEN = 50.^16^ Host reads were detected by aligning against the hg19 human reference genome using Bowtie 2 and then removed.^17^ Microbial abundance at the species level was calculated from the quality-filtered metagenomes using MetaPhlAn3 based on over one million clade-specific marker genes.^18^ ARGs were predicted from the metagenomes using a deep learning approach, DeepARG, with short_reads_pipeline.^19^ ARGs were also predicted using a traditional, alignment-based approach, ShortBRED,^20^ against the Comprehensive Antibiotic Resistance Database (CARD) v3.2.5 using UniRef90 as the background database and the non-default parameter --clustid = 0.95.^6^^,^^21^^,^^22^

### Alpha and beta diversity analysis

Microbial and ARG feature tables were imported into QIIME2 2020.11.^23^ Alpha and beta diversity metrics as well as principal coordinate analysis (PCoA) plots were generated using q2-diversity. Alpha diversity metrics computed included the number of observed features and Shannon diversity, whereas Bray–Curtis dissimilarity was used for beta diversity estimation. For each of the travel-related variable, the changes in the alpha and beta diversity of the gut microbiome and resistome after travel were compared among groups using pairwise-differences and pairwise-distances in q2-longitudinal, respectively.^24^

### Metagenomic binning

Metagenomic binning was performed using three different algorithms, namely MetaBAT 2,^25^ MaxBin 2 and CONCOCT.^26^^,^^27^ An optimized, non-redundant set of bins was then calculated using DAS Tool for each sample.^28^ The bins were further refined using RefineM,^29^ quality-checked using CheckM and taxonomically classified using GTDB-Tk.^30^^,^^31^ Potential microbial hosts of ARGs were then inferred by searching the ARGs against the final bins using tblastn.

### Statistical analysis

Differences between paired samples were tested using the Wilcoxon Signed-Rank test, whereas differences between non-paired samples were tested using the Kruskal–Wallis or Mann–Whitney test. Differences in beta diversity were tested using permutational multivariate analysis of variance (PERMANOVA) with 9999 permutations using the adonis function in q2-diversity. For variables found to be significantly associated with the changes in the alpha or beta diversity of the faecal microbiome or resistome after travel as detected by q2-longitudinal, linear mixed effects (LME) models were built using q2-longitudinal. Travel-related variables, their interactions with time (travel), sex and age were used as fixed effects, whereas subject ID was used as a random effect. For the analysis of beta diversity, the first coordinate (PC1) of PCoA was used as the input metric. Differentially abundant microbial species and ARGs were identified using LEfSe with the default threshold at linear discriminant analysis (LDA) score > 2, unless specified otherwise.^32^ Changes in the prevalence of microbes and ARGs were tested using Fisher’s exact test. *P*-values were adjusted using the Benjamini–Hochberg procedure to control for multiple comparisons. Differences were considered statistically significant when *P* < 0.05 or *q* < 0.1.

## Results

### Characteristics of the study cohort

A total of 110 healthy Chinese travellers from Hong Kong were recruited for this project. After exclusion of subjects with recent antibiotic use, 90 subjects were included in this study. This study cohort had a median age of 21 years (range: 19–65 years) and was mostly female (68.9%) (Table S1). The majority of the participants was travelling on vacation (42.2%) or for volunteer or missionary purposes (38.9%). Southeast (SE) Asia (41.1%) and East Asia (24.4%) were the most common travel destinations, and the median duration of travel was 13 days (range: 4–94 days). About one-fourth (24.4%) and 16.7% of the participants used alcohol-based hand sanitizer (ABHS) or doxycycline as antimalarial prophylaxis during travel, respectively. None of the subjects consumed other antibiotics during travel.

### International travel does not significantly alter the faecal microbiome or resistome

**Figure 1:**
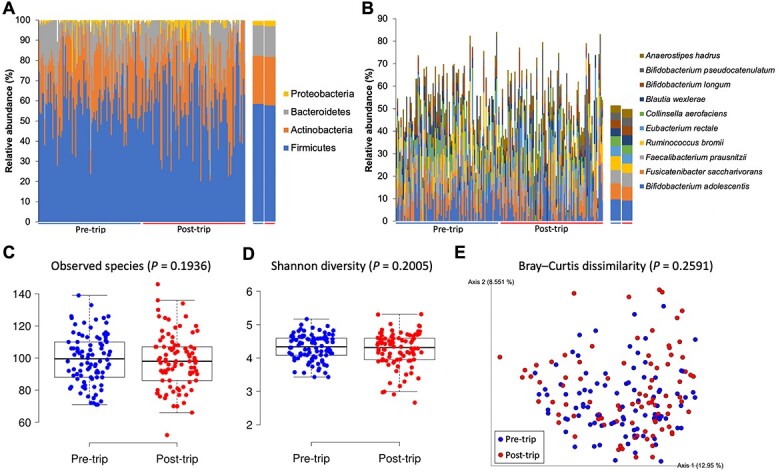
Faecal microbiome of travellers before and after travel. Taxonomic composition of the top four most abundant bacterial phyla (A) and top 10 most abundant bacterial species (B) in the faecal microbiome. The left panels show the results of each individual, whereas the right panels show the averaged values. Alpha diversity estimated using the number of observed species (C) and Shannon diversity index (D). (E) PCoA plot of beta diversity based on Bray–Curtis dissimilarity. *P*-values in alpha and beta diversity analysis were calculated based on the Wilcoxon Signed-Rank test and PERMANOVA, respectively.

An average of 21 491 900 quality-filtered non-human reads were obtained for each of the 180 stool samples. The baseline (pre-trip) faecal microbiota of the travellers was dominated by the bacterial phyla Firmicutes (58.3%), Actinobacteria (23.8%) and Bacteroidetes (15.2%) (Figure 1A) and the species *Bifidobacterium adolescentis* (9.5%), *Fusicatenibacter saccharivorans* (7.2%), *Ruminococcus bromii* (6.2%) and *Faecalibacterium prausnitzii* (5.9%) (Figure 1B). Travel did not significantly alter the taxonomic composition, alpha or beta diversity of the faecal microbiome (*P* > 0.05) (Figure 1). In particular, travel did not significantly change the relative abundance (pre-trip: 1.8%, post-trip: 2.2%) or prevalence (pre-trip: 90.0%, post-trip: 96.7%) of the family Enterobacteriaceae (*P* > 0.05). However, culture-based screening for MDR bacteria revealed that the carriage rate of ESBL-E in the stool specimens increased significantly from 52.8 (47/89) to 72.4% (63/87) after return of travel (*P* = 0.0083). CPE was only detected in one post-trip specimen.

**Figure 2:**
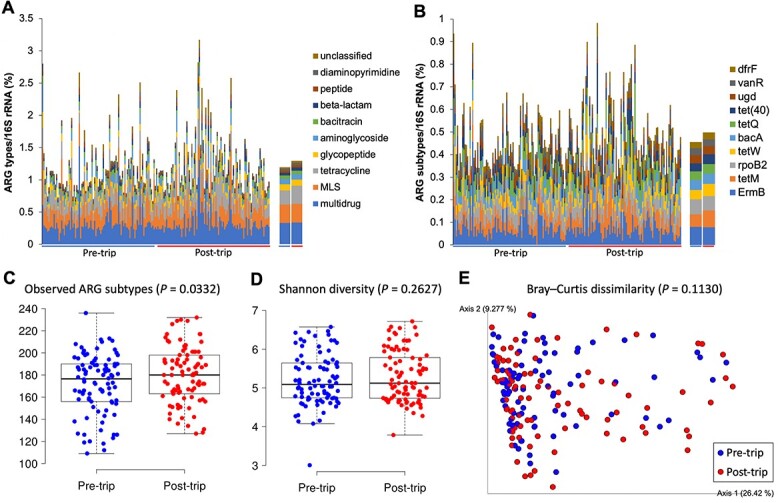
Faecal resistome of travellers before and after travel. Relative abundance of the top 10 most abundant ARG types (A) and subtypes (B) normalized to the 16S rRNA gene. The left panels show the results of each individual, whereas the right panels show the averaged values. Alpha diversity estimated using the observed number of ARG subtypes (C) and Shannon diversity index (D). (E) PCoA plot of beta diversity based on Bray–Curtis dissimilarity of ARG subtypes. *P*-values in alpha and beta diversity analysis were calculated based on the Wilcoxon Signed-Rank test and PERMANOVA, respectively.

DeepARG and ShortBRED with CARD predicted a total of 441 and 295 ARGs from all samples, respectively (Data Sets S1 and S2). A recent benchmarking study suggested a superior performance of DeepARG over ShortBRED in shotgun metagenomics-based ARG prediction, and therefore, ARGs predicted with DeepARG were used in subsequent analysis.^33^ A total of 32 ARG types (classes) and 403 ARG subtypes were predicted from the baseline faecal metagenomes of the travellers. The baseline faecal resistome comprised an average of 1.2% of the metagenome and was dominated by multidrug (0.3%), macrolide-lincosamide-streptogramin (0.3%) and tetracycline (0.2%) ARG types (Figure 2A). An average of 172 ARG subtypes were detected in the baseline faecal resistome of the participants (Figure 2C), with *ermB* (0.08%), *rpoB2* (0.07%) and *tetM* (0.06%) being the most abundant members (Figure 2B). Similar to the case of the faecal microbiome, travel did not significantly alter the taxonomic composition of the dominant ARG subtypes nor the alpha (Shannon) or beta diversity of the faecal resistome (*P* > 0.05); however, travel significantly increased the number of observed ARG subtypes (*P* = 0.0332) (Figure 2). Phenotypic ESBL positivity was positively associated with the predicted relative abundance of individual major ESBL families (TEM, SHV, CTX-M and OXA) (*P* < 0.01) and total ESBLs (*P* < 0.0001).

### International travel results in enrichment and acquisition of ARGs in stool

**Figure 3:**
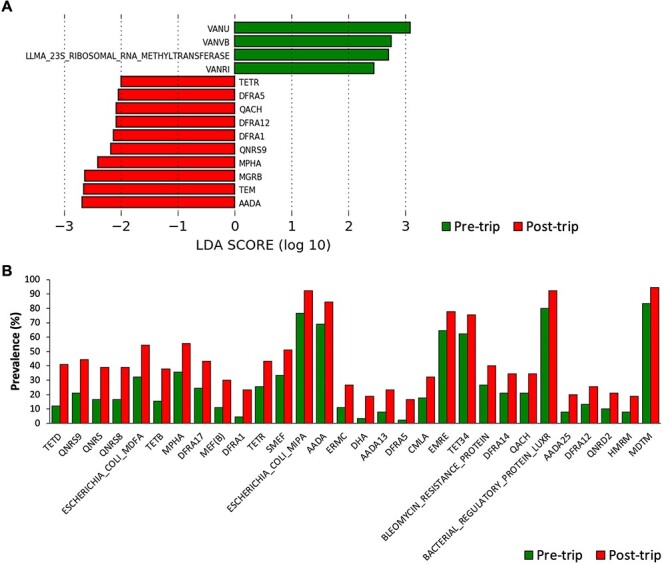
Enrichment and acquisition of ARGs after travel. (A) Differentially abundant ARG subtypes in pre- and post-trip stools as detected by LEfSe. (B) ARG subtypes that were significantly more prevalent in post-trip stools (*q* < 0.1).

Although travel did not significantly alter the taxonomic composition of the dominant ARG subtypes, LEfSe analysis revealed the enrichment of 10 rare ARG subtypes (relative abundance < 0.01%) post-trip, including *aadA*, TEM, *mgrB*, *mphA* and *qnrS9* (Figure 3A). We then performed metagenomic binning analysis to elucidate the potential microbial origins of these ARGs. Results showed that eight of the enriched ARG subtypes were detected in bins belonging to *Escherichia/Shigella flexneri* from the post-trip samples (Table S2), suggesting an important role of this species in the acquisition of these ARGs during travel. Thirty ARG subtypes significantly increased in prevalence after travel (*q* < 0.1) (Figure 3B). The largest changes were observed in *tetD* (12.2 to 41.1%), *qnrS9* (21.1 to 44.4%), *qnrS* (16.7 to 38.9%), *qnrS8* (16.7 to 38.9%), *Escherichia coli mdfA* (32.2 to 54.4%) and *tetB* (15.6 to 37.8%) (Table S3). Most ARGs enriched in relative abundance post-trip also increased in prevalence after travel, except for TEM and *mgrB*.

### Changes in alpha and/or beta diversity of faecal microbiome and resistome are associated with travel-related variables

We then examined the effects of 14 travel-related variables (Table S1) on the changes in the alpha and beta diversity of their faecal microbiome and resistome after travel. Results showed that none of the variables affected the changes in the alpha diversity of the faecal microbiome (*P* > 0.05) (Table S4). In contrast, five variables significantly affected the changes in the beta diversity of the faecal microbiome, including destination grouped according to income (*P* = 0.0009, *q* = 0.0042) or region (*P* = 0.0098, *q* = 0.0343), travel purpose (*P* = 0.0197, *q* = 0.0552) and use of ABHS (*P* = 0.0001, *q* = 0.0007) or doxycycline during travel (*P* = 0.0001, *q* = 0.0007) (Figure S1, Table S5). Notably, travel to low- or middle-income countries, Africa or SE Asia, or use of ABHS or doxycycline during travel resulted in significantly larger changes in the beta diversity of the faecal microbiome. Travel to low- or middle-income countries (*P* = 0.0154) or SE Asia (*P* = 0.0002) was also significantly associated with the acquisition of ESBL-E based on culture test (Table S5). However, none of these variables showed a significant effect in LME models, which also took into account potential confounding covariates including sex, age and subject ID (Table S6).

**Figure 4:**
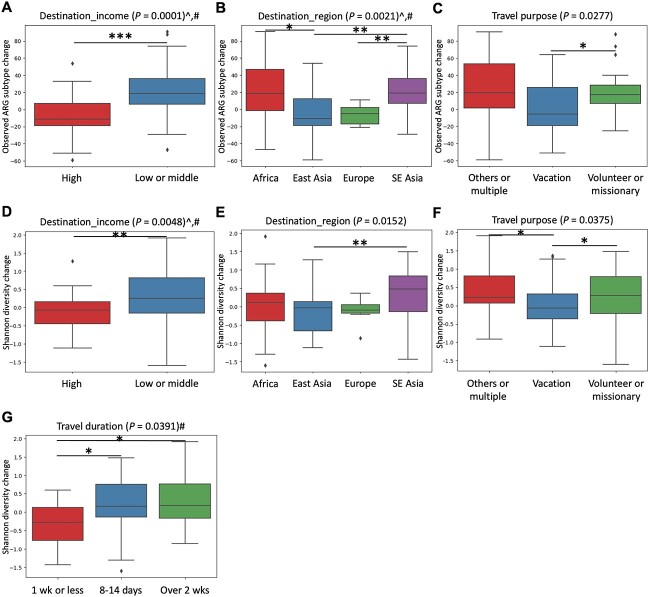
Significant travel-related variables affecting the changes in the alpha diversity of the faecal resistome after travel based on the observed number of ARG subtypes (A–C) and Shannon diversity index (D–G). Variables that remained significant after controlling for age and sex in LME models were marked with the # symbol. *P*-values were calculated based on the Kruskal–Wallis test. ^*^P < 0.01, ^*^^*^P < 0.05, ^*^^*^^*^P < 0.001, ^*q* < 0.1.

Unlike the case of the faecal microbiome, three travel-related variables significantly affected the changes in the number of observed ARG subtypes, including destination_income (*P* = 0.0001, *q* = 0.0014), destination_region (*P* = 0.0021, *q* = 0.0147) and travel purpose (*P* = 0.0277, *q* = 0.1293) (Figure 4, Table S4). These three variables and travel duration also significantly affected the changes in the Shannon diversity of ARG subtypes (destination_income: *P* = 0.0048, *q* = 0.0672; destination_region: *P* = 0.0152, *q* = 0.1064; travel purpose: *P* = 0.0375, *q* = 0.1369; travel duration: *P* = 0.0391, *q* = 0.1369). Notably, travel to low- or middle-income countries, Africa or SE Asia, or a longer travel duration of more than seven days resulted in significantly larger changes in the alpha diversity of the faecal resistome. Except for travel purpose, all these variables remained significant for at least one of the alpha diversity indices in LME models (Table S7).

**Figure 5:**
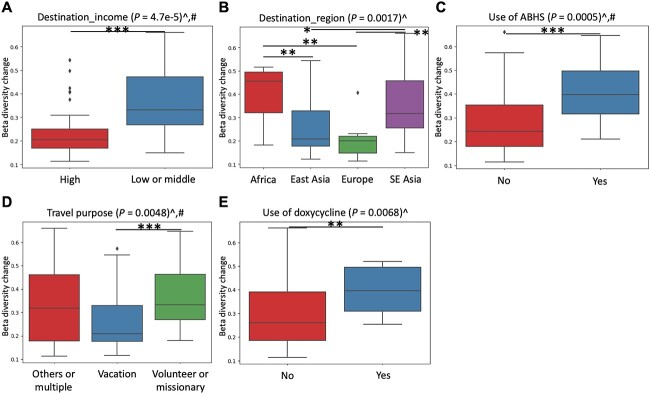
Significant travel-related variables affecting the changes in the beta diversity of the faecal resistome after travel based on Bray–Curtis dissimilarity of ARG subtypes. Variables that remained significant after controlling for age and sex in LME models were marked with the # symbol. *P*-values were calculated based on the Kruskal–Wallis test. ABHS, alcohol-based hand sanitizer. ^*^P < 0.01, ^*^^*^P < 0.05, ^*^^*^^*^P < 0.001, ^*q* < 0.1.

The same five variables that significantly affected the changes in the beta diversity of the faecal microbiome also affected those of the faecal resistome (destination_income: *P* = 4.70E-05, *q* = 0.0007; destination_region: *P* = 0.0017, *q* = 0.0079; travel purpose: *P* = 0.0048, *q* = 0.0168; use of ABHS: *P* = 0.0005, *q* = 0.0035; use of doxycycline: *P* = 0.0068, *q* = 0.0190) (Figure 5, Table S4). Destination_income, travel purpose and use of ABHS remained significant in LME models (Table S7). LEfSe analysis revealed the enrichment of multiple tetracycline ARGs, including *tetM*, *tetW*, *tetO*, *tetA*, *tetP*, *tetC* and *tetR*, in travellers who used the tetracycline-class antibiotic doxycycline during travel (Table S8). The full lists of ARGs and microbial species enriched pre- and post-trip for each subgroup of the five variables are provided in Tables S8 and S9, respectively.

## Discussion

Our results showed that travel did not significantly alter the taxonomic composition of the dominant ARGs; however, the relative abundances of 10 low abundance ARGs were enriched post-trip. This echoes the results of a Swedish study, in which only nine rare ARGs were enriched after travel,^7^ and suggests that international travel can alter the abundances of the rare ARGs while leaving the dominant ARGs largely unaffected. Most ARGs enriched post-trip in our study were detected in bins of *Escherichia/S. flexneri* from the post-trip samples, suggesting this species as an important source of these ARGs acquired during travel. *Escherichia/S. flexneri* can be found in the environment, food and untreated water. Therefore, it is tempting to speculate that travellers acquired ARG-bearing *Escherichia/S. flexneri* via contaminated environment or food during travel. The prevalence of 30 ARGs increased in our study cohort after travel, with a rate of increase of at least 13% (and up to 6.5-fold). A recent study also reported significant acquisition of 56 ARGs in Dutch travellers after travel to countries in Asia and Africa.^6^ These findings support an important role of international travel in ARG acquisition.

We showed that destination grouped according to income or region significantly affected the changes in the beta diversity of the gut microbiome and the alpha and beta diversity of the gut resistome, with travel to low- or middle-income countries, Africa or SE Asia, resulted in significantly larger changes in all diversity indices. In contrast to our finding, the only other study that has examined the effect of travel destination on the gut microbiome did not observe any significant influence of the visited region (Asia, Sub-Saharan Africa or Latin America) on the beta diversity of the gut microbiome in French travellers.^9^ The discrepancy may be due to a small sample size in the French study (*n* = 39) and/or differences in the travel destinations. The effects of travel destination on the gut resistome have been extensively characterized in a recent study on Dutch travellers.^6^ They revealed that travellers to SE Asia had the largest increase in the resistome alpha diversity, a finding in agreement with our observation. AMR rates in low- and middle-income countries, including those in SE Asia, are generally high,^34^ possibly due to country-specific veterinary and human antibiotic use.^35^

We showed that ABHS use during travel significantly changed the gut microbiome and resistome composition. ABHS use during travel led to an overall net gain of ARGs (Table S8), albeit an insignificant effect on the overall alpha diversity. The use of ABHS and the frequency of ABHS use during travel have been reported to alter the gut microbiome composition.^12^ Besides, the use of ABHS has been reported to facilitate the growth of MDR strains of *Acinetobacter baumannii*.^36^ Taken together, these findings highlight the possible collateral effect of excessive ABHS use on the development and spread of antibiotic-resistant bacteria and ARGs during travel.

The use of doxycycline during travel also significantly changed the gut microbiome and resistome composition and enriched multiple tetracycline ARGs. Doxycycline is commonly used by travellers as antimalarial prophylaxis during travel to countries where there is high risk of malaria transmission, such as those in Africa and South America. This drug is taken 1–2 days before travel, daily during travel and for 28 days after return from trip.^37^ As the post-trip stool samples in this study were collected within 1–2 days after return from travel and the median travel duration is 13 days, our results suggest that use of the antibiotic for a median period of 2 weeks during travel is enough to enrich ARGs, a finding that may have practical implications on the use of doxycycline, and possibly other antibiotics, as antimalarial prophylaxis during travel. Future studies on the impact of gut resistome by the use of other forms of antimalarial prophylaxis with minimal or reduced spectrum of antibacterial activity would be preferred.

The normal gut microbiota is capable to protect against the invasion and/or outgrowth of indigenous and foreign microorganisms via a process called colonization resistance.^38^ Consumption of antibiotics like doxycycline causes an imbalance (dysbiosis) in the gut microbiota, opening up niches for the colonization and/or outgrowth of competing species, including AMR bacteria, and possibly resulting in the changes observed in the gut microbiome and resistome composition. The use of ABHS is expected to affect the hand skin microbiota in a similar manner; however, how this potentially alters the gut microbiome/resistome in this context is unclear and warrants further research.

We found that a longer travel duration resulted in significantly larger changes in the alpha diversity of the gut resistome. A significant but weak effect of travel duration on the acquisition of ARGs was also reported in a previous study.^6^ These results agree with the intuition that a longer stay would increase the chance of encounter with and thus acquisition of new ARGs.

We observed significant associations between travel to low- or middle-income countries or SE Asia and ESBL-E acquisition based on culture test. A high number of acquisitions of ESBL-E has also been reported among European travellers who travelled to southern Asia,^2^ and more recently Asian travellers who travelled to lower income countries.^39^ By providing data from a population not previously well described, we have strengthened the role of travel to high-prevalence areas in the acquisition of ESBL-E. Future research should focus on the impact of ESBL-E acquisition on individual travellers and the communities to which they return.^40^

There are several limitations to our study. First, all participants were university staff or students and therefore do not represent the general population in terms of socioeconomic status nor ethnic Chinese from the mainland. Second, most metadata collected in this study were self-reported. Third, stool samples from travellers while abroad were not collected. Lastly, no non-travel controls were included in this study. Nevertheless, our study has elucidated the effects of travel and specific travel-related risk factors on the entire communities of microbes and ARGs in the gut and provided insights into the potential risk factors for the acquisition/enrichment of ARGs during international travel. Our findings highlighted current practice related to travel in the context of antimicrobial resistance, particularly advocating the use of alternatives to doxycycline as antimalarial prophylaxis.

## Supplementary Material

Suppl_figure_taad027

Suppl_tables_new_taad027

Suppl_data_sets_taad027

## Data Availability

Raw shotgun metagenomic data generated from this study were deposited in the NCBI Sequence Read Archive (http://www.ncbi.nlm.nih.gov/sra) under BioProject accession PRJNA834885.

## CRediT author Statement

Man Kit Cheung (Formal analysis-Lead, Visualization-Lead, Writing—original draft-Lead), Rita Ng (Writing—review and editing-Supporting), Christopher Lai (Writing—review & editing-Supporting), Chendi Zhu (Writing—review and editing-Supporting), Eva Au (Resources-Lead), Jennifer Yau (Investigation-Lead), Carmen Li (Writing—review and editing-Supporting), Ho Cheong Wong (Resources-Supporting), Bonnie Wong (Conceptualization-Equal), Kin On Kwok (Conceptualization-Equal, Funding acquisition-Supporting), Zigui Chen (Formal analysis-Supporting), Paul Chan (Conceptualization-Equal), Grace Lui (Conceptualization-Lead, Funding acquisition-Lead, Methodology-Lead, Resources-Lead, Supervision-Lead, Writing—review and editing-Lead), Margaret Ip (Conceptualization-Lead, Funding acquisition-Lead, Methodology-Lead, Supervision-Lead, Writing—review and editing-Lead).
