# Supplementary material for: Alterations in faecal microbiome and resistome in Chinese international travellers: a metagenomic analysis
Source: J Travel Med. 2023 Mar 2;30(6):taad027. doi: 10.1093/jtm/taad027 (PMC10628765; doi:10.1093/jtm/taad027)
Supplement: Suppl_figure_taad027 [file suppl_figure_taad027.pdf]

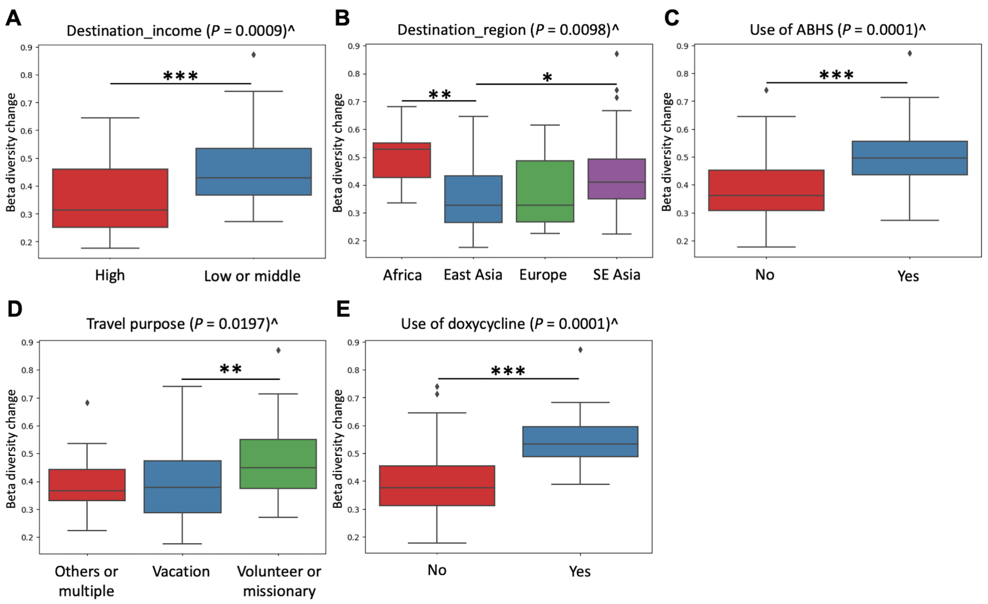

Figure S1. Significant travel-related variables affecting the changes in the beta diversity of the fecal microbiome after travel based on Bray–Curtis dissimilarity. *P* values were calculated based on Kruskal–Wallis test. ABHS, alcohol-based hand sanitizer. *$P < 0.01$, **$P < 0.05$, ***$P < 0.001$, ^$q < 0.1$. None of the variables remained significant after controlling for age and sex in LME models.
